# Effectiveness and safety of Tai Chi for chronic pain of knee osteoarthritis: A protocol for systematic review and meta-analysis

**DOI:** 10.1097/MD.0000000000028497

**Published:** 2022-01-14

**Authors:** Guangxin Guo, Boyi Wu, Shengji Xie, Jianghan Xu, Xu Zhou, Guanghui Wu, Ping Lu

**Affiliations:** a School of Acupuncture-moxibustion and Tuina, Shanghai University of Traditional Chinese Medicine, Shanghai, China; b Shanghai Municipal Hospital of Traditional Chinese Medicine, Shanghai University of Traditional Chinese Medicine, Shanghai, China.; c Yueyang Hospital of Integrated Traditional Chinese and Western Medicine, Shanghai University of Traditional Chinese Medicine, Shanghai, China.

**Keywords:** chronic pain, knee osteoarthritis, meta-analysis, protocol, Tai Chi

## Abstract

**Background:**

Chronic pain (CP) has been a major area of interest in the field of knee osteoarthritis (KOA), further aggravating the dysthymia, stiffness, and dysfunction of KOA patients. As an important part of complementary and alternative medicine, Tai Chi has a positive effect on KOA patients. The systematic review is to evaluate the effectiveness and safety of Tai Chi for KOA patients with CP.

**Methods:**

A systematic search will be performed in the following electronic databases for randomized controlled trials to evaluate the effectiveness and safety of Tai Chi in treating chronic pain of knee osteoarthritis: the Cochrane Library, PubMed, EMBASE, OVID-MEDLINE, and four Chinese databases (Wan Fang, CNKI, CBMdisc and VIP). Each database will be searched from inception to Dec. 2021. The process will include study selection, data extraction, risk of bias assessment and meta-analysis.

**Results:**

This proposed study will evaluate the effectiveness and safety of Tai Chi for KOA patients with CP. Improvement in pain and adverse effects of KOA will be included in our measure.

**Conclusions:**

This proposed systematic review and meta-analysis will evaluate the existing evidence on the effectiveness and safety of Tai Chi for KOA patients with CP.

**Dissemination and ethics:**

The results of this review will be disseminated through peer-reviewed publication. This review does not require ethical approval because all of the data used in this systematic review and meta-analysis have already been published. Furthermore, all of these data will be analyzed anonymously during the review process.

**INPLASY registration number:**

INPLASY2021120020.

## 1 Introduction

Chronic pain (CP) is one of the most important symptoms of knee osteoarthritis (KOA), further leading to the gradual deterioration of the stiffness, dysfunction, emotional disorder and poor quality of life.^[1,2]^ CPKOA is a greatest contributor to disability globally and bring a serious burden on healthcare systems.^[3–5]^ As a important factor affecting patients’ repeated visits and even their choice of surgery, CP is defined as pain lasting more than three months or longer than normal tissue healing time,^[6]^ and negatively impact psychological well-being and social connectedness and of patients.^[1]^ CP in KOA patients results in decreased physical mobility, muscle atrophy, anxiety, and depression.^[7,8]^ Long-term CP can respectively induce plasticity of the central nervous system and seriously affect the physical and mental health of KOA patients.^[9,10]^ Unfortunately, there is still no cure for the progress of CPKOA. Current mainstream therapies have some limitations, such as long-term use of drugs maybe lead to side effects, and surgical treatments can cause greater trauma. A study based on the data from the UK General Practice Research Database showed that TKR's lifetime risk increased from 2.9% to 10.6% and from 1.8% to 7.7% for men between 1991 and 2006.^[11]^ Therefore, it is particularly important to find effective and safe treatment methods to relieve the pain of CPKOA and reduce the medical burden.

According to the Guideline of Osteoarthritis Research Society International (OARSI), physical exercise is positive for individuals with KOA.^[12]^ As an important part of complementary and alternative medicine, Tai Chi is easier accepted by KOA patients with its advantages of simple, safe, convenient and economical, small space occupation and less load on joints.^[13]^ Tai Chi is widely used to treat CP conditions, Tai Chi is widely used to treat CP diseases, such as knee osteoarthritis and fibromyalgia syndrome.^[14]^ Studies have shown that Tai Chi has produced beneficial effects for treating KOA, such as reducing pain, improving physical function, reducing depressed mood and improving quality of life.^[8,15–17]^ The analgesic effect of Tai Chi on CPKOA has been confirmed by an increasing number of reports. The analgesic effect of Tai Chi on CPKOA has been confirmed by more and more reports,^[14,18]^ thus showing the application prospect of Tai Chi in the clinical practice of CPKOA.

Meta-analysis, an important tool for evidence-based medicine, is a powerful widely accepted statistical technique.^[19]^ To date, there is no valid evidence on the follow-up effects of Tai Chi for CPKOA. Compared with other active therapies, there is still insufficient evidence to support or refute the value of Tai Chi in the treatment of CPKOA. Despite the advantages of non-invasive and relatively low cost and the growing popularity of Tai Chi compared to other therapies, discussions continue regarding the effectiveness of Tai Chi in complementary and alternative medicine.^[14,20]^ Therefore, we perform this protocol to comprehensively evaluate the effect of Tai Chi on CPKOA.

## 2 Methods

### 2.1 Study registration

This protocol was registered on the International Platform of Registered Systematic Review and Meta-analysis Protocols on Dec. 14, 2021 (registration number:). We will strictly perform this protocol by following the Preferred Reporting Items for Systematic Reviews and Meta-analysis Protocol statement guidelines.^[21]^

### 2.2 Inclusion criteria for study selection

#### 2.2.1 Type of studies

Only randomized controlled trials about Tai Chi for CPKOA will be included, with language restrictions in English or Chinese. Case report, experience report, and laboratory studies will not be included.

#### 2.2.2 Types of participants

All patients with CPKOA will be included without limitation of age, race, gender, economic level, and severity. CPKOA patients have pain that lasts longer than 3 months.

#### 2.2.3 Types of interventions

##### 2.2.3.1 Experimental interventions

The intervention in the experimental group included Tai Chi exercise compared with the control group. There will be no limitation on the methods, duration, and frequency of Tai Chi training.

##### 2.2.3.2 Control interventions

The interventions of control group will involve any therapy other than Tai Chi (e.g., medication, placebo, routine care, other sports and exercises etc.).

#### 2.2.4 Types of outcome measures

##### 2.2.4.1 Primary outcomes

Primary outcomes include pain, such as visual analog scale, the Western Ontario and McMaster Universities Arthritis Index (WOMAC) pain etc..

##### 2.2.4.2 Additional outcomes

(1) WOMAC stiffness;
(2) WOMAC physical function;
(3) A 12-item Short-Form Health Survey (SF-12);
(4) Pressure pain threshold;
(5) Adverse events.

### 2.3 Search strategy

We will perform a comprehensive search in the Cochrane Library, PubMed, EMBASE, OVID-MEDLINE, and four Chinese databases (Wan Fang, CNKI, CBMdisc and VIP) for articles published before Dec. 2021. Only randomized controlled trials that used Tai Chi as the main treatment for adults with CPKOA will be included. The Chinese and English search strategies in PubMed database are shown in Table 1. The search terms in the Chinese databases have the same meaning as those used in the English databases. There will be no language restrictions in this review.

**Table 1:** Search terms used in Pubmed database.

Search strategy
#1 Intervention: ((((((((((((((Taiqi) OR (Taichi Chuan)) OR (t’ai chi chuan)) OR (shadowboxing)) OR (Tai-ji)) OR (Tai Chi)) OR (Chi, Tai)) OR (Tai Ji Quan)) OR (Ji Quan, Tai)) OR (Quan, Tai Ji)) OR (Taiji)) OR (Taijiquan)) OR (T’ai Chi)) OR (Tai Chi Chuan)) OR (“Tai Ji”[Mesh])
#2 Participant: ((((Knee Osteoarthritides) OR (Knee Osteoarthritis)) OR (Osteoarthritis of Knee)) OR (Osteoarthritis of the Knee)) OR (“Osteoarthritis, Knee”[Mesh])
#3 Study design: (randomized controlled trial [pt] OR controlled clinical trial [pt] OR randomized [tiab] OR placebo [tiab] OR clinical trials as topic [mesh: noexp] OR randomly [tiab] OR trial [ti])NOT (animals [mh] NOT humans [mh])
#4 #1 AND #2 AND #3

### 2.4 Identification of studies

**Figure 1:**
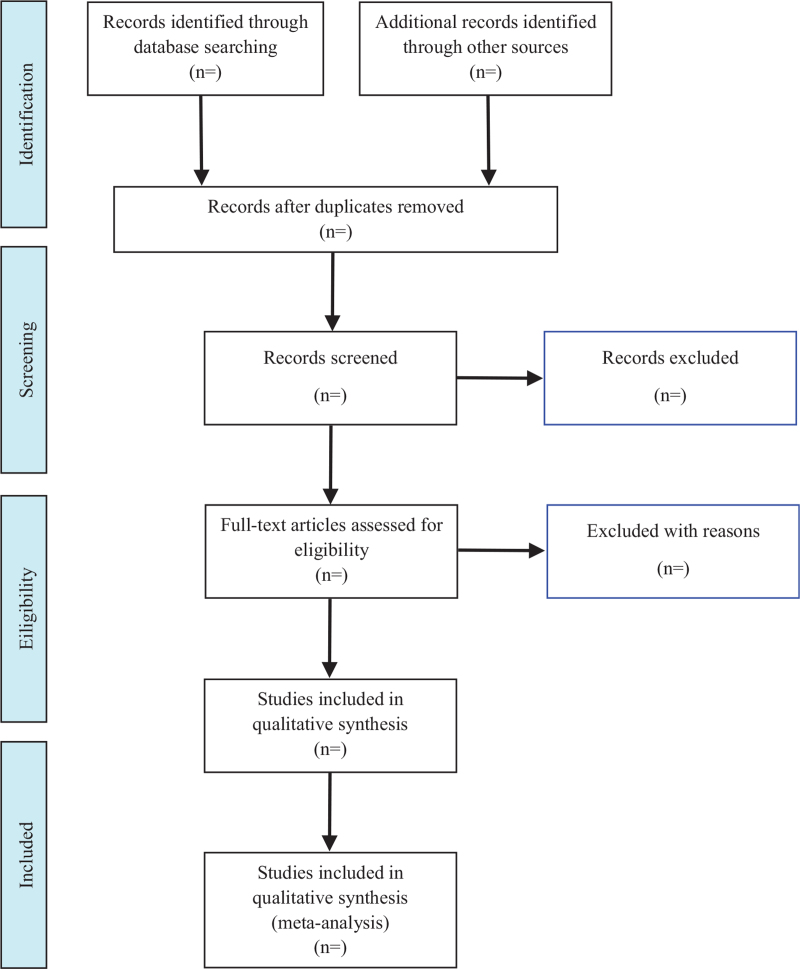
Flow diagram of study selection.

All the search results will be imported into EndNote software (V.x9) for management. Two reviewers (SX and BW) will independently screen all potentially eligible studies. Titles and abstracts will be screened first to exclude irrelevant citations. Full text of all the articles with potentially relevant abstracts will be retrieved and screened according to the study eligibility criteria. Disagreements will be resolved by consensus or discussion with a third reviewer (GG). The research flow chart is shown in Figure 1.

### 2.5 Data collection

Two reviewers will extract data from the included literature through Microsoft Excel 2010 (Microsoft company, Seattle), mainly including the following information (Table 2):

(1) General information about the study, such as authors, year of publication, country, groups, sample size, age, and gender;
(2) Detailed treatment information, such as diagnostic criteria and parameters of intervention;
(3) Pain scores. Other outcome measurements, such as SF-12 or WOMAC stiffness, WOMAC physical function etc., will be extracted if they are mentioned in the study.

**Table 2:** Data extraction form.

First authors	Year	Country	Sample size	Mean age
Gender	Pain location	Duration	Follow-up	Diagnostic criteria
Experimental group intervention	Control group intervention	Main outcome assessments		

### 2.6 Quality of evidence assessment

Based on Grading of Recommendations Assessment Development and Evaluation, we will assess the quality of evidence as 4 grades: high quality, moderate quality, low quality and very low quality.^[22]^ In addition, we will use the online guide development tool to carry out this process. Any differences will be resolved by obtaining the consensus of all reviewers.

### 2.7 Risk of bias assessment

Study quality will be assessed in RevMan V5.4 (the Nordic Cochrane Centre, Cochrane Collaboration)^[23]^ using the Cochrane risk of bias tool. The risk of bias for each of the following domains will be assessed for each study: (1) random sequence generation, (2) allocation concealment, (3) blinding of participants and personnel, (4) blinding of outcome assessments, (5) incomplete outcome data, (6) selective reporting, and (7) other bias. Each study included will be rated as having a high, low, or unclear risk of bias. Two reviewers (SX and BW) will evaluate the consistency of all the extracted data and quality ratings. Disagreements will be resolved by discussion with a third reviewer (GG).

### 2.8 Statistical analysis

Revman 5.4 software will be used to perform statistical analysis. For discontinuous variables, the risk ratio with 95% confidence interval (CI) will be selected. For continuous variables, the weighted mean difference with 95% CI will be selected when the measuring instruments are the same, and the standardized mean difference with 95% CI will be selected when the measuring instruments are different. We will use the fixed-effect model if there is no significant heterogeneity (*P* > .1 or *I*^*2*^ < 50%). If there is a significant heterogeneity (*P* > .1 or *I*^*2*^ < 50%), we will conduct subgroup analysis or sensitivity analysis to identify possible causes of heterogeneity among populations.

### 2.9 Subgroup analysis

If the necessary data are available, subgroup analysis will be conducted according to the following criteria:^[24]^

(1) The treatment period;
(2) Different styles of Tai Chi (e.g., Chen style, Sun style, Yang style).

### 2.10 Subgroup analysis

To identify the robustness of the meta-analysis, low-quality trials, with high risks of bias or outcomes that are seriously distant from the rest of the data, will be excluded.

### 2.11 Ethics and dissemination

Ethical approval will not be in need because the data used in this systematic review will not be individual patient data, and there will be no concerns regarding privacy.

## 3 Discussion

CP is a major symptom of KOA, and CPKOA patients often require long-term use of non-steroidal anti-inflammatory drugs, other drugs, and even choose surgery for pain relief. However, the pain of CPKOA is still not completely resolved for a long time.

Tai Chi can improve balance, muscle strength, cardiopulmonary function and sleep quality, relieve depression, anxiety and tension and other psychological problems, bringing beneficial effects to KOA individuals.^[25–27]^ Tai Chi has a holistic view consistent with the TCM diagnosis and treatment thinking, focusing on the overall recuperation and function recovery of the human body.^[16,28,29]^ A study found that long-term regular Tai Chi exercise can restore the senesthesia sensitivity of the elderly, and the improvement effect of knee joint and ankle joint proprioception is better than running and swimming.^[30]^ Some studies strongly recommend 20-weeks Sun style Tai Chi exercise program (three times a week, 20–40 minutes per time) to relieve the pain and improve physical function of KOA.^[17,31]^ Among them, the relief of bone pain may be related to the exercise intervention of bone stimulation, often accompanied by the participation of some cytokines or minerals.^[32,33]^ A study based on the fNIRS system found that an exercise program 3 times a week for 6 weeks significantly reduced functional activity in the dorsolateral prefrontal cortex (DLPFC) in patients with CPKOA,^[10]^ and provided evidence about the pain modulatory effects of exercise at DLPFC level which is correlated with clinical improvements in CPKOA patients. Long-term Tai Chi practice is also benefit to the brain white matter.^[34]^

Although many studies have reported the effectiveness of these supplementary interventions, there is a general lack of determination of the most effective therapeutic Tai Chi methods in the field of CPKOA. Therefore, this online meta-analysis will provide a detailed summary and analysis of the latest evidence, with a focus on available Tai Chi methods. We hope that our findings will help patients, clinicians, and healthcare policy makers make better treatment choices for CPKOA.

## Author contributions

GG, SX, and BW conceived the study. JX, XZ, GW and PL provided general guidance to the drafting of the protocol. SX and GG drafted the protocol. SX designed the search strategy. GG and SX drafted the manuscript. JX, XZ, BW, GW and PL reviewed and revised the manuscript. All authors have read and approved the final version of the manuscript.

**Funding acquisition:** Guangxin Guo, Ping Lu.

**Writing – original draft:** Guangxin Guo, Boyi Wu, Shengji Xie, Jianghan Xu, Xu Zhou, Guanghui Wu, Ping Lu.

**Writing – review & editing:** Guangxin Guo, Ping Lu.
